# Retired Nurses Can Improve Retention in Prevention of Mother-to-Child Transmission Programmes

**DOI:** 10.24248/EAHRJ-D-19-00011

**Published:** 2019-11-29

**Authors:** Zahra Persson Theilgaard, Mercy G Chiduo, Leo Flamholc, Jan Gerstoft, Ib C Bygbjerg, Martha Moshi Lemnge, Terese L Katzenstein

**Affiliations:** a Department of Infectious Diseases, Copenhagen University Hospital, Rigshospitalet, Copenhagen, Denmark; b National Institute for Medical Research, Tanga Centre, Tanga, Tanzania; c Department of Infectious Diseases, University Hospital of Malmö, Malmö, Sweden; d Department of Clinical Medicine, University of Copenhagen, Copenhagen, Denmark; e Department of Public Health, University of Copenhagen, Copenhagen, Denmark

## Abstract

**Background:**

The success of prevention of mother-to-child transmission (PMTCT) programmes depends on retention of mothers throughout the PMTCT cascade.

**Methods:**

In a clinical trial of short-course combination antiretroviral therapy (cART) for PMTCT in Tanzania, senior nurses were employed to reduce the substantial loss-to-follow up (LTFU) rate.

**Results:**

Following intervention, the relative risk (RR) of receiving a CD4 count result and antiretroviral therapy was 1.16 (95% confidence interval [CI], 1.05 to 1.27), the RR of delivery at clinic was 2.51 (95% CI, 2.06 to 3.06), the RR for reporting for follow-up at 6 to 8 weeks postpartum was 4.63 (95% CI, 3.41 to 6.27), and the RR for being retained until 9 months postpartum was 28.19 (95% CI, 11.81 to 67.28). No significant impact on transmission was found.

**Conclusion:**

Significantly higher retention was found after senior nurses were employed. No impact on transmission was found. Relatively low transmission was found in both study arms.

## INTRODUCTION

The World Health Organization (WHO) and the global community have made a commitment to eliminating mother-to-child transmission (MTCT) of HIV as a public health problem.^1^ This can be achieved through combination antiretroviral therapy (cART) initiated during pregnancy (for women not already receiving cART) and continued throughout the breastfeeding period.^2^ Since November 2015, the WHO has recommended that cART initiated during pregnancy be continued lifelong worldwide.^3^ This strategy has been termed “Option B+”. Retention of HIV-infected women—keeping the women in antiretroviral therapy programmes throughout the cascade of diagnosis, treatment, and follow-up— is fundamental to the success of Option B+. Successful follow-up is at least until the end of the breastfeeding period to prevent MTCT and lifelong to ensure maternal health. Impediments, including fear of stigmatisation, long waiting times at the cART clinics, and transportation costs, may deter pregnant women from enrolling and adhering to cART programmes, including prevention of MTCT (PMTCT) programmes,^4–6^ leading to low retention rates, even in the Option B+ era.^7^

In Tanzania, PMTCT services are integrated with antenatal care services at the mother and child health (MCH) clinics. All pregnant women who come for the first antenatal care visit, receive group pretest HIV counselling, are tested for HIV, and given individual post-test counselling during which the result of their HIV test is conveyed. Currently, women identified as HIV-infected start cART regardless of CD4 count as soon as possible after diagnosis, in line with the global WHO strategy, whereby all who test positive for HIV must begin cART as soon as possible.^8,9^

The data presented here are from the ComTru study, a clinical trial conducted from 2006 to 2011 comparing 2 short-course antiretroviral regimens for PMTCT in the pre-B+ era (https://clinicaltrials.gov/ct2/show/NCT00346567). During the period when the ComTru study was carried out, the guideline still differentiated between individuals who had advanced HIV (CD4<200 cells/mm^3^ regardless of clinical stage, or stage 3 disease plus CD4<350 cells/mm,^3^ or clinical stage 4 regardless of CD4 cell count) and individuals who did not. Women who had advanced HIV were referred immediately to the designated HIV clinic (the care and treatment clinic) to begin lifelong cART.^10^

Women who did not have advanced HIV remained enrolled in the MCH clinic and were given the PMTCT antiretroviral regimen. Until 2008, the PMTCT regimen recommended by the National Tanzanian guidelines was composed of single-dose nevirapine (sd-NVP) for the mother and sd-NVP for the infant.

From 2008, the PMTCT antiretroviral regimen was as outlined below.

Mother:

- Antepartum from 28 weeks gestational age: zidovudine 300 mg twice daily
- Intrapartum: sd-NVP 200 mg + zidovudine 300 mg every 3 hours and lamivudine 150 mg every 12 hours until delivery
- Postpartum: zidovudine/lamivudine (300 mg/150 mg) twice daily for 7 days.

Infant:

- sd-NVP (2 mg/kg) within 72 hours of birth + zidovudine (4 mg/kg) twice daily for 7 days, if the mother had received antepartum zidovudine for at least 4 weeks, or 28 days, if the mother had received zidovudine for less than 4 weeks antepartum.

The need to differentiate between advanced and early-stage HIV meant that women found to be HIV-infected had to have their CD4 cell counts determined and await the result of this test before they knew which antiretroviral regimen they were eligible for and where they would receive HIV-related care. Women who continued to receive care at the MCH clinic and began antepartum zidovudine were expected to come monthly for drug refills.

Infants were given cotrimoxazole until they ceased breastfeeding and subsequently received HIV-negative test results. HIV-infected infants continued to receive cotrimoxazole and cART. DNA polymerase chain reaction (PCR)-based early infant diagnosis of HIV at age 4 to 6 weeks of age began in 2010. Final antibody-based diagnosis was made at age 18 months.

ComTru study participants were HIV-infected women enrolled during pregnancy and followed until 9 months post-partum. A significant loss to follow-up of study participants was found within the first months of the study (Figure 1). To reverse this trend, 3 experienced senior nurses (age >55) were employed by the ComTru study as study nurses to provide extended individual counselling sessions to pregnant women newly diagnosed with HIV, and to see the ComTru study participants at clinic visits. The nurses where put in charge of each study site. These nurses were chosen for their extensive experience, including experience working in an MCH clinic setting. They all lived in Tanga. Furthermore, because they had been planning retirement, engaging them in the ComTru study did not drain the pool of nurses, who were actively employed and were needed at the clinics and hospitals.

In this paper we present data on the retention of women at key study visits before and after the employment of senior study nurses, as well as MTCT rates in the 2 study arms, indicating the achieved level of adherence to study medication.

## METHODS

### Study Design and Participants

The ComTru study was a randomised open-label clinical trial (ClinicalTrials.gov, ID number NCT00346567) conducted from June 2006 through April 2011 in Tanga, Tanzania. Study sites were Bombo Regional Hospital, Ngamiani Primary Health Centre and Markorora Primary Health Centre. Bombo Regional Hospital is a large hospital with 600 beds serving the whole of Tanga region with a population of approximately 2.1 million. At the time of the ComTru study, its MCH clinic had a catchment area with a population of 5,739 (2012 population census).^11^ Makorora and Ngamiani Primary Health Centres still had active MCH clinics and serve populations of approximately 16,664 and 16,303 people respectively (2012 population census).^11^ Makorora Primary Health Centre was only included as a site from 2008.

The sample size calculation was based on the estimated occurrence of NNRTI resistance at 6 to 8 weeks postpartum and on the premises of noninferiority. Using the 9% NNRTI resistance-rate after intrapartum sd-NVP and postpartum AZT/3TC found in the TOPS study^12^ an initial sample size of 269 patients was needed to detect a 10% difference between the arms with 80% power and a 2.5% 1-sided significance level when anticipating a 4% loss to follow-up (LTFU). During the first 6 months, LTFU was much larger than anticipated and the sample size calculation was repeated with an anticipated LFTU of 35%, yielding a sample size of 397 patients. As we had once been taken aback by a larger LTFU than anticipated, the total sample size was further increased to 566 to safeguard the primary outcome.

Pregnant women were tested for HIV during their first antenatal care (ANC) visit.^10^ HIV infected women received information about the study during post-test counselling and were included if they were >18 years of age, were ART naïve, agreed to deliver at 1 of the study clinics, to return for follow-up and, from March 2008, to provide contact information to enable tracing. Exclusion criteria included fulfilling the national criteria for cART at the time: CD4<200 (before 2008) or CD4 <350 10^6^/l (after 2008) and WHO clinical stage 3, creati-nine >1.5 mg/dl, ALAT >140 U/l or systemic disease requiring medical treatment. From September 2008, after introduction of zidovudine, women with haemoglobin less than 7.5 g/dl were also excluded.^10^

### Counselling and Clinic Visits

Women enrolled in 2006 and 2007 (Group 1) were seen at all clinic visits by clinic nurses employed by the health centres or hospital.

Women enrolled in 2008 to 2010 (Group 2) were given pretest counselling by clinic nurses, however all subsequent counselling and clinic visits were with the senior study nurse, who was designated to taking care of the study participants at that clinic or hospital. This included post-test counselling, during which the study participant would be given the option to have the senior study nurse escort her home or within sight of her home to facilitate support and tracing. The study participants and senior study nurse exchanged contact telephone numbers and the study participants were encouraged to contact their senior study nurse when in need, including for support, if necessary, at the time of delivery and if they needed to contact a health-care facility outside the scheduled study visits.

ART for PMTCT was provided at 28 weeks of gestational age or as soon as possible thereafter and women came for drug refill every 4 weeks. After delivery, women were seen on day 7, day 28, week 6 to 8 and at 9 months postpartum. Children born to study participants were tested for HIV at the 6 to 8 weeks and 9 months postpartum visits. If study participants absconded from clinic visits, their senior study nurse would trace them, at first through a phone call and if this did not ensure that the participant reported to the clinic, the nurse would attempt to trace the participant to their home.

Assessment of Retention The study cohort was divided into women enrolled in 2006 and 2007 before the employment of senior study nurses (Group 1) and those enrolled from 2008 through 2010 after the employment of senior study nurses (Group 2). For each of the 2 groups the following proportions were calculated:

1. The proportion of women who came to collect their study drugs
2. The proportion of women who delivered at the study clinic or came to the clinic following delivery
3. The proportion of women who were retained in the study at 6 to 8 weeks postpartum
4. The proportion of women who were retained in the study at 9 months postpartum

### ComTru Study Antiretroviral Therapy Arms

Eligible women were randomised 1:1 by computer generated block randomisation to receive either sd-NVP (200 mg) intrapartum with AZT/3TC (300 mg/150 mg) intrapartum (they received AZT every 3 hours and 3TC every 12 hours until delivery^10^) and twice daily for 7 days postpartum or intrapartum sd-NVP combined sd-tenofovir/emtricitabine (300 mg/200 mg). All infants were given sd-NVP (2 mg/kg) as soon as possible and within 72 hours of birth. From September 2008 women also received antepartum AZT (300 mg) twice daily from 28 weeks of gestational age or as soon as possible thereafter and infants received AZT (4 mg/kg) twice daily for 7 or 28 days in accordance with the WHO guidelines^13^ and updated Tanzanian guidelines.^18^ Women enrolled from June 2006 to April 2007 were given the option to use infant feeding formula provided free of charge. Women enrolled after April 2007 were counselled to breastfeed exclusively for 6 months.^14^

### Assessment of Children's HIV Status

Whole blood samples were collected from the children at 6 to 8 weeks, 3 months, and 9 months of age. If whole blood samples could not be obtained, heel prick filter paper samples were obtained (Whatman 3 Qualitative Circles, Whatman, Kent, UK or S&S 903 filter cards, Schleicher & Schuell, Dassel, Germany).

Initial assessment of the children's HIV status was done at the National Institute for Medical Research (NIMR), Tanga Centre by ultra-sensitive HDB 24 antigen analysis (Perkin Elmer Life Sciences, Boston, MA, USA) using the Perkin Elmer NEK 050 Alliance^®^ HIV-p24 antigen kit and the Perkin Elmer NEP 116 ELAST^®^ Elisa amplification system as described by Schupbach et al.^15,16^

If a positive p24 antigen result was found a subsequent sample was analysed. Two positive p24 results prompted immediate referral of the child to an HIV clinic

Samples were stored at −80°C and shipped to Denmark for final assessment of HIV status using HIV RNA PCR COBAS^®^ Ampliprep/COBAS^®^ TaqMan HIV-1 Test, version 1.0 (Roche Molecular Systems, Inc., Branchburg, NJ, USA) or the Roche Amplicor HIV-1 Monitor Test version 1.5 (F. Hoffmann-La Roche, Basel, Switzerland) in accordance with the manufacturer's instructions. Infants with HIV RNA<100 copies/ml were classified as HIV-uninfected, while infants with HIV RNA levels >10,000 cp/ml were classified as HIV-infected and samples containing 100 to 10,000 cp/ml were considered inconclusive. A subsequent sample was analysed to confirm or disprove HIV positive and inconclusive samples when possible.^17^

When insufficient plasma was available for HIV RNA PCR, DNA PCR was carried out on filter paper samples using the qualitative AMPLICOR^®^ HIV-1 DNA Test, version 1.5 (Roche Molecular Systems, Inc., Branchburg, NJ, USA). DNA was extracted from the filter paper as described by Sherman et al.^18^

### Statistical Analyses

Statistical analyses were done using STATA version 10 (StataCorp LLC, Texas, USA). Categorical variables were analysed by Fisher's exact test and by computing the relative risk (RR) and its confidence intervals (CI). Continuous variables were analysed by calculating and comparing means using a t-test.

### Ethical Considerations

Ethical approval for the ComTru study was given by the Medical Research Coordination Committee (MRCC) of the Tanzanian National Institute for Medical Research (Reference NlMRlHQIR.8alVol.00436) and consultative approval was granted by the Development-Country Committee of the Danish National Committee on Biomedical Research Ethics. Participants were provided with oral and written information in Kiswahilli about the study background and procedures for mothers and children. Mothers gave written consent to participate in procedures related to themselves and their children. Consent forms were likewise in Kiswahili.

## RESULTS

### Baseline Characteristics

566 women enrolled into the ComTru study, 221 in Group 1 and 345 in Group 2. The baseline characteristics were generally well balanced between the 2 groups. The only significant differences were employment and marital status (Table 1). Of the 345 women in Group 2, 198 (57%) were enrolled at Ngamiani Primary Health Centre, 80 (23%) were enrolled at Makorora Primary Health Centre and 67 (19%) were enrolled at Bombo Regional Hospital. As 1 nurse was stationed at each site, the nurse-to-study participant ratio was different at the 3 sites.

**TABLE 1.** Comparison of Baseline Characteristics of Women Enrolled in the ComTru Study Prior to Hiring Senior Nurses (Group 1) Compared to Women Enrolled After Hiring Senior Study Nurses (Group 2)

Characteristic	Group 1 (2006–2007) (n=221)		Group 2 (2008–2011) (n=345)		*P* Value
	Total	n (%)	Total	n (%)	
**Mean age±SD, years**	221	27.8±5.5	345	27.5±5.0	.49
**Religion**	220		344		
Christian		46 (21%)		66 (19%)	.67
Muslim		174 (79%)		278 (81%)	
**Education**	216		344		
Primary		176 (81%)		299 (87%)	.11
Secondary		26 (12%)		34 (10%)	
College/university				1 (0%)	
None		14 (6%)		10 (3%)	
**Job**	220		344		
Housewife		159 (72%)		280 (81%)	.01
Employed		22 (10%)		15 (4%)	
Self-employed		33 (15%)		40 (12%)	
Jobless		6 (3%)		5 (1%)	
Other		0 (0%)		4 (1%)	
**Marital status**	220		339		
Single		37 (17%)		30 (9%)	.002
Married		169 (77%)		277 (82%)	
Widowed		2 (1%)		5 (1%)	
Divorced		7 (3%)		4 (1%)	
Cohabiting		5 (2%)		23 (7%)	
**Partner's mean age±SD**	191	35.4±7.5	323	34.6±8.3	.32
**Partner's educational level**	217		344		.50
Primary		149 (69%)		236 (69%)	
Secondary		61 (28%)		89 (26%)	
College		3 (1%)		12 (3%)	
Other		1 (0%)		4 (1%)	
None		3 (1%)		3 (1%)	
**Partner's job**	218		343		.39
Peasant		5 (2%)		6 (2%)	
Employed		82 (38%)		109 (32%)	
Self-employed		129 (59%)		353 (65%)	
Jobless		1 (0%)		0 (0%)	
Student		1 (0%)		2 (1%)	
Other		0 (0%)		2 (1%)	
**Present pregnancy**					
Mean gravidity±SD	221	2.9±1.6	345	2.7±1.3	.17
Mean parity±SD	221	1.7±1.4	345	1.6±1.3	.25
Mean gestational age±SD	219	23.9±8.5	345	23.5±7.4	.64
Mean CD4 count±SD, cells/μl	221	556±612	345	490±669	.24

Abbreviation: SD, standard deviation

### Patient Retention

Overall 116 (20%) of the study participants never collected their study drugs and only 293 (52%) brought their children for HIV testing at 6 to 8 weeks postpartum.

Women who were LTFU differed significantly from the women, who reported to the clinic at 6 to 8 weeks post-partum in the following ways: They were slightly younger (mean age of those who reported for week 6 to 8 was 28.1±5.1 years, while the mean age of those LTFU before week 6 to 8 was 27.0±5.3 years (*P*=.01) and more often single (9% vs. 15% *P*=.04). No statistically significant difference was found in any other baseline characteristics between the 2 groups.

Retention-rates among women enrolled before hiring senior study nurses (Group 1) and after (Group 2) are shown in Figure 1. As shown in Table 2, significant increases in retention were found in Group 2 compared to Group 1 (*P*<.001) at all time-points.

**TABLE 2.** Retention in the ComTru Study Following Employment of Senior Study Nurses (Group 2) Compared With Before Employment of Senior Study Nurses (Group 1)

Time Point	Relative Risk of Retention in Group 2 Relative to Group 1	95% Confidence Interval
**Collecting antiretroviral therapy**	1.16	1.05–1.27
**Delivering at 1 of the study clinics or coming to the clinic after delivery**	2.51	2.06–3.06
**Retention in study at 6–8 weeks postpartum**	4.63	3.41–6.27
**Retention in study at 9 months postpartum**	28.19	11.81–67.28

### Mother-to-Child Transmission

Based on the test results of 299 children tested at 6 to 8 weeks postpartum from whom at least 1 sample was tested by HIV RNA PCR, HIV DNA PCR or p24 antigen analysis and on 236 children tested at 9 months postpartum from whom at least 1 sample was tested by HIV RNA PCR, HIV DNA PCR or p24 antigen analysis, no significant difference in MTCT was found between the 2 ComTru study arms: Among children age 6 to 8 weeks HIV prevalence was 5.8% (9/156) and 5.6% (8/143) in the AZT/3TC and TDF/FTC arm respectively (RR 1.0; 95% CI, 0.4 to 2.4; *P*=.9). Of these children, 197 were born to mothers who had received antepartum AZT and 102 were born to mothers who had not. The overall MTCT-rate among children whose mothers had received antepartum AZT was 3.6% versus 9.8% among the children whose mothers had not (RR 0.36; 95% CI, 0.14 to 0.92; *P*=.03). Among children age 9 months HIV prevalence was 9.5% (11/116) and 11.7% (14/120) in the AZT/3TC and TDF/FTC arm respectively (RR 1.2; 95% CI, 0.6 to 2.6; *P=*.6). Of these children, 171 were born to mothers who had received antepartum AZT and 65 were born to mothers who had not. The overall MTCT-rate among children whose mothers had received antepartum AZT was 7.6% versus 18.5% among children whose mothers and not (RR 0.4; 95% CI, 0.2 to 0.9; *P*=.02).

No significant difference in HIV prevalence was found between children of mothers in Group 1 (none of whom received ante-partum AZT) compared to the children of women in Group 2, who did not receive ante-partum AZT: at age 6 to 8 weeks HIV prevalence was 8.1% (3/37) and 10.8% (7/65) in Group 1 and Group 2 respectively (RR 1.4; 95% CI 0.4 to 4.8; *P*=.7), and at age 9 months HIV prevalence was 21.4% (3/14) and 19.2% (10/52) in Group 1 and Group 2 respectively (RR 0.9; 95% CI, 0.3 to 2-8; *P*=.9).

## DISCUSSION

The main findings in this study were significantly lower LTFU after introduction of senior study nurses and relatively low MTCT-rates in both short-term PMTCT study arms with no significant difference between Group 1 and Group 2 or between treatment arms. Our MTCT rates may be underestimated because the mother–child pairs who were LTFU might have had a higher MTCT rate than the mother–child pairs who reported for follow-up.

The transmission rates at 6 to 8 weeks were in line with other studies using similar regimens.^1,19^ Furthermore, the significant effect of antepartum AZT on MTCT rates reiterates results from previous studies.^20,21^ MTCT is preventable.^22^ With effective combination therapy during the latter part of pregnancy and treatment of the infant, even for very short periods, combined with no breast-feeding, the MTCT-rate can be reduced to <2%.^23–26^ Therefore, there is hope that widespread implementation of option B+ can curb MTCT. However, substantial difficulties with referral and retention of women for continuous antepartum and postpartum treatment have been reported.^6,27–32^ A systematic review of studies of retention in care in the B+ era in Africa found retention rates of 79%, 75% and 69% after 6, 12 and 24 months follow-up respectively in pooled analyses.^33^ However, data from Malawi, where B+ was first implemented, have shown no significant increase in pregnant women initiating ART following implementation of option B+ compared to before.^7^ Furthermore, the same study found that more women withdrew from PMTCT services or transferred out after the implementation of B+ compared to before.^7^ Another study from Malawi examining retention of women beginning cART after implementation of the B+ programme, found that women beginning antiretroviral therapy while pregnant were 5 times more likely to fail to return to clinic after their first visit than women beginning cART for their own health.^34^

A recent study of retention in care during the first year of the B+ programme in Tanzania found that 2 years after enrolment into the B+ programme, 59% of women were LTFU, and nearly one-third of those lost, were lost during pregnancy.^35^

A qualitative study carried out among women referred for life-long antiretroviral therapy from the ComTru study indicated that among the main deterrents from reporting were fear of stigmatisation and conflicts with work resulting from frequent clinic visits and potentially resulting in loss of income. These deterrents have also been found to affect clinic attendance among women receiving option B+ in South Africa.^36^ Fear of stigmatisation may also have deterred the women from reporting for HIV-related care at the RCH.^6^

Qualitative data from health-care personnel and pregnant and postpartum women from Malawi have indicated that barriers to beginning ART on the same day as diagnosis and to retention in care include inadequate time for counselling and support in order to come to terms with the diagnosis and begin and remain in lifelong treatment.^37^ Supportive counselling and support from health-care personnel was found to greatly assist in overcoming the fear of stigmatisation and disclosure of their HIV status and to provide motivation to seek cART among women from the ComTru study.^4^ Thorough individual counselling was highlighted as a recommendation to ensure that those, who do not seek treatment, are motivated to do so.^4^

The implementation of B+ has led to an increased work load to be lifted by nurses in MCH clinics and thus to a need to expand the workforce.^38,39^ However this expansion is in many settings lagging behind the increase in patient up-take leading to overburdened, overwhelmed nurses who are unable to give the time, attention and counselling needed by newly diagnosed women living with HIV.^40–43^ Employing senior nurses in this capacity provides an opportunity for them to continue working under less taxing conditions, while making use of the expertise and experience they possess for the good of these vulnerable patients.

Employment of senior study nurses as familiar contact persons for HIV infected women attending RCH clinics may have worked by reducing the fear of stigmatisation through empowering counseling and by giving the HIV-infected women a feeling of being accepted and cared for despite their HIV diagnosis. However, further studies would be needed to corroborate this hypothesis.

### Limitations

The limitations of the data on retired nurses presented in this paper, include that the ComTru study was not designed specifically to investigate the impact of hiring senior nurses, and that potential confounders include increased awareness in the community of HIV and HIV related interventions as well as changes in PMTCT regimens. However, the significant improvement in retention rates found along with the reports from study participants of the support and courage derived from their interaction with the senior study nurses, warrant further exploring of the impact that employing nurses, who would otherwise have retired, as counsellors in the clinics may have. The main limitation of the data on MTCT was the large loss to follow-up as well as the changes in guidelines that happened over the course of the study. However efforts were made as described to compensate for these factors.

## CONCLUSION

Our findings combined with the worrying shortage of health-care personnel in resource-constrained settings^42,43^ support that retaining able-minded senior nurses as counsellors could potentially alleviate the burden on overworked health-care personnel and increase retention in life-long cART among HIV infected women during and after pregnancy. However, further studies are needed to assess this strategy in the B+ era outside a clinical trial setting.

Additionally, identification of where women, who forgo antenatal and postpartum HIV-related care and treatment, give birth, could provide an opportunity to reduce the risk of MTCT by providing ART at the time of delivery. Because of the high risk of NNRTI resistance following sd-NVP^44–46^ alternative short-course strategies for PMTCT with less risk of NNRTI resistance development were sought and could still be warranted.
